# PD129 Extension Of An Influenza Vaccination Program To Include Those Aged 50 To 64 Years: A Rapid Health Technology Assessment Approach

**DOI:** 10.1017/S0266462324003660

**Published:** 2025-01-07

**Authors:** Karen Cardwell, Carol Mc Loughlin, Dana Swanton, Patricia Harrington, Conor Teljeur, Máirín Ryan

## Abstract

**Introduction:**

Influenza causes considerable morbidity and mortality. Vaccine effectiveness is variable due to the evolution of influenza viruses and antigenic mismatch. In Ireland, influenza vaccination is reimbursed for all individuals aged at least 65 years as well as those who are at increased risk of exposure or severe disease. However, it is unclear whether reimbursement should be extended from 2023 to 2024 to those aged 50 to 64 years in the general population.

**Methods:**

A rapid health technology assessment (HTA) format was chosen due to time constraints, as the findings were to inform a decision for the upcoming influenza season only and would not result in a permanent change to the vaccination program. National sentinel surveillance data (for seasons 2010-2011 to 2022-2023) and hospitalization data for the publicly funded healthcare system (for years 2010-2022) were used. Central Statistics Office data indicated that the projected population for 2023 of those aged 50 to 64 years was 914,379. Given the one-year timeframe, the economic evaluation was limited to a costing analysis to estimate the potential cost associated with expanding the program.

**Results:**

Surveillance data showed year-on-year variability in influenza incidence. Excluding the years not considered representative due to the influence of COVID-19, on average 12.9 percent (range 9 to 17%) of notified cases, 11.8 percent (range 9 to 17%) of influenza-related hospital admissions, 23.2 percent (range 12 to 34%) of influenza-related intensive care admissions, and 9.6 percent (range 0 to 44%) of influenza-related deaths per year were in people aged 50 to 64 years. Data were not disaggregated according to vaccination or risk status. The estimated mean incremental cost of extending eligibility for the 2023 to 2024 season (based on a 35% uptake rate) was EUR2.27 million (range EUR1.9 million to EUR2.65 million, depending on vaccine costs).

**Conclusions:**

Given the relatively modest number of influenza-related hospitalizations in the 50- to 64-year age group, and the substantial year-on-year variability in vaccine effectiveness, the potential for a reduction in demand for hospital care is likely to be small. While a rapid HTA approach facilitates timely decision support, it creates challenges for exploring the more complex facets of an intervention.

